# The Influence of Metastable Cellular Structure on Deformation Behavior in Laser Additively Manufactured 316L Stainless Steel

**DOI:** 10.3390/nano11112859

**Published:** 2021-10-26

**Authors:** Na Li, Zhengyang Li, Yujie Wei

**Affiliations:** 1 LNM, Institute of Mechanics, Chinese Academy of Sciences, Beijing 100190, China; lina@imech.ac.cn (N.L.); zyli@imech.ac.cn (Z.L.); 2 School of Engineering Sciences, University of Chinese Academy of Sciences, Beijing 100049, China

**Keywords:** laser additive manufacturing, metastable cellular structures, 316L stainless steel, coherent precipitates, stacking fault energy

## Abstract

Metastable cellular structures (MCSs) play a crucial role for the mechanical performance in concentrated alloys during non-equilibrium solidification process. In this paper, typifying the heterogeneous 316L stainless steel by laser additive manufacturing (LAM) process, we examine the microstructures in cellular interiors and cellular boundaries in detail, and reveal the interactions of dislocations and twins with cellular boundaries. Highly ordered coherent precipitates present along the cellular boundary, resulting from spinodal decomposition by local chemical fluctuation. The co-existences of precipitates and high density of tangled dislocations at cellular boundaries serve as walls for extra hardening. Furthermore, local chemical fluctuation in MCSs inducing variation in stacking fault energy is another important factor for ductility enhancement. These findings shed light on possible routines to further alter nanostructures, including precipitates and dislocation structures, by tailoring local chemistry in MCSs during LAM.

## 1. Introduction

Laser additive manufacturing (LAM) is an emerging approach to achieve tailored structural components with more exceptional performances than conventional processing routines [1,2,3,4]. The near one-step process leads to enhanced mechanical properties, including high yield strength, intermediate tensile ductility, and better fatigue resistance. From the microstructural aspect, typical metastable cellular structures (MCSs) resulted from LAM process play a central role for high strength and intermediate-to-high ductility in many LAM materials, e.g., 316L stainless steel (SS) [1,2], Al-based alloys [4,5] and Ni-based superalloys [6,7]. During LAM process, the intrinsic solidification features—including fast cooling rate of 10^4^~10^6^ K/s and repeated thermal passes lead to redistribution of local chemical composition during solidification, and consequentially prevailing MCSs far from well-equilibrated state. In another case of high (medium)-entropy alloys, with four or more principal elements, the sluggish solutes diffusion during non-equilibrium solidification promotes to form MCSs as well [8,9,10]. Usually, accompanied with the formation of MCSs, there are plenty of dislocations, solutes segregation, and precipitates, which collectively contribute to a uniquely heterogeneous nature of MCSs. It is therefore expected that MCSs with chemical and structural heterogeneity would bring forth distinct deformation behavior and could be utilized for better tailoring a strength–ductility combination.

Taking 316L SS as an example, intensive efforts have been devoted to the intrinsic feature of MCSs [2,11,12,13,14]. For instance, Morris et al. [2] reported that MCSs are endowed with elemental segregations, a high density of dislocations and Mn-enriched SiO_2_ precipitates along the cellular boundary. Other works revealed MCSs are embedded with Cr or Mo-Cr enriched precipitates [15]. Bertsch et al. [14] found that a MCS has micro-segregation networks superimposed on dislocation structures without observable precipitates. Indeed, the formation of MCSs in LAM alloy depend on chemical segregations of solid–liquid interface during solidification. At the advancing solid–liquid interface, insufficient solute diffusion leads to the imbalance in solubility, and consequently micro-segregation occurs in concentrated alloys [13]. The segregation plays a critical role in precipitation process and heterogeneous chemical distributions at MCSs [16,17,18]. Theoretical studies suggest that the spinodal decomposition depends on the magnitude and species of the solutes enrichment by segregation at free surface [19,20]. Therefore, understanding the origins of precipitation process induced by spinodal decomposition is beneficial to the tailored chemical configuration at MCSs.

Additionally, how combined features in MCSs contribute to the desirable mechanical property in LAM process is of practical importance. MCSs have been reported to be able to facilitate deformation twinning to transfer through the adjacent cells, and give rise to progressive work-hardening [21,22]. Although the twinning mechanism has been commonly highlighted [2,22,23], the detail interactions of MCSs with the twin boundary is still not fully exploited. Another important feature associated with chemical fluctuation in MCSs is the stacking fault energy (SFE): In company with local chemical fluctuation in MCSs, SFE changes. It has been well known that SFE plays a key role for plastic deformation, as broadly demonstrated in TRIP and TWIP steels [23], high entropy alloys [24], and so on [25,26]. Thus, evaluating the SFE evolution at MCSs may supply further insights into deformation mechanisms.

To summarize, both characteristics about MCSs and their correlation with deformation process are critical for further optimization of heterogeneous structures in LAM metallic alloys. Following these incentives, we examine prominent microstructures in cellular interiors and MCSs of as-built 316L SS in detail, and demonstrate how chemical fluctuations and the existence of heterogeneous walls may boost deformability and hardenability of this particular LAM alloy.

## 2. Materials and Methods

The gas-atomized 316L SS powders, in weight composition 18.18% Cr, 11.36% Ni, 2.27% Mo, 0.97% Si, 0.06% C, and Fe balance, were characterized by the ICP-MS and used for the LAM process. A five-axis handling system combining with a fiber-coupled diode laser system YDFL-2000-CW-MM (Feibo Laser Technologies Co. Ltd, Shanghai, China) with a wavelength of 1080 nm was utilized for printing. Argon was chosen as the shielding and carrier gas to avoid oxidation and feed powders during manufacturing. The laser beam was focused on the 316L SS substrate to create a melting pool, and when injected powders were melted, a deposited layer is subsequently formed after solidification. A unidirectional scan strategy with 45° rotation in each layer was employed. A scanning speed of 24 mm/s and the laser power of 1200 W were applied during deposition. In the successive track, the in-plane shift spacing was set to be 0.75 mm with a hatching angle 45° in the longitudinal direction (LD). A constant height offset of 0.5 mm (plane to plane) was used in the build direction (BD). These process parameters were chosen based on extensive process optimization so as to obtain fully dense samples [27]. Samples with an overall dimension of 50 × 50 × 10 mm were built on a 316L SS substrate under the processing conditions.

For microstructure characterizations, the as-built samples were sectioned along the LD-BD plane. The sectioned samples were then analyzed using a SEM (FEI-600) equipped with an electron backscattered diffraction (EBSD) detector (Oxford Instruments, Tubney Wood, UK) [28,29]. In our EBSD analyses, we adopted an accelerating voltage of 20 kV and a probe current of 2.9 nA. We interpreted the raw EBSD data for geometrically necessary dislocations (GNDs) density evolutions by the Channel 5 and Aztec (Oxford Instruments HKL, Abingdon, UK) software. We followed the method from the literatures [30,31] to relate local misorientations with GND density. It involves the following steps: (1) We obtain an EBSD image of the sample under consideration; (2) the kernel average misorientation (KAM) map is then calculated from the raw EBSD data, from which we obtain the average misorientation between every pixel and a kernel of surrounding pixels; (3) we use the following equation to calculate GND density ρ: $\rho =\frac{\alpha \theta }{Xb}$, where *α* = 2, *θ* is the local misorientation angle, *X* is the step size for KAM map and *b* is the magnitude of the Burgers vector.

Tensile tests were performed using a Instron 5967 testing system at a constant strain rate 10^−3^ s^−1^. Dog-bone specimens with a gauge length 20 mm were prepared for tensile test. To identify the possible deformation mechanisms, post-mortem deformed samples were examined using a transmission electron microscopy (TEM, JEM-2100F) at an accelerating voltage of 200 kV. We follow Du et al. [32] to abstract lattice distortion using the geometric phase analysis (GPA) approach.

## 3. Results and Discussion

**Figure 1:**
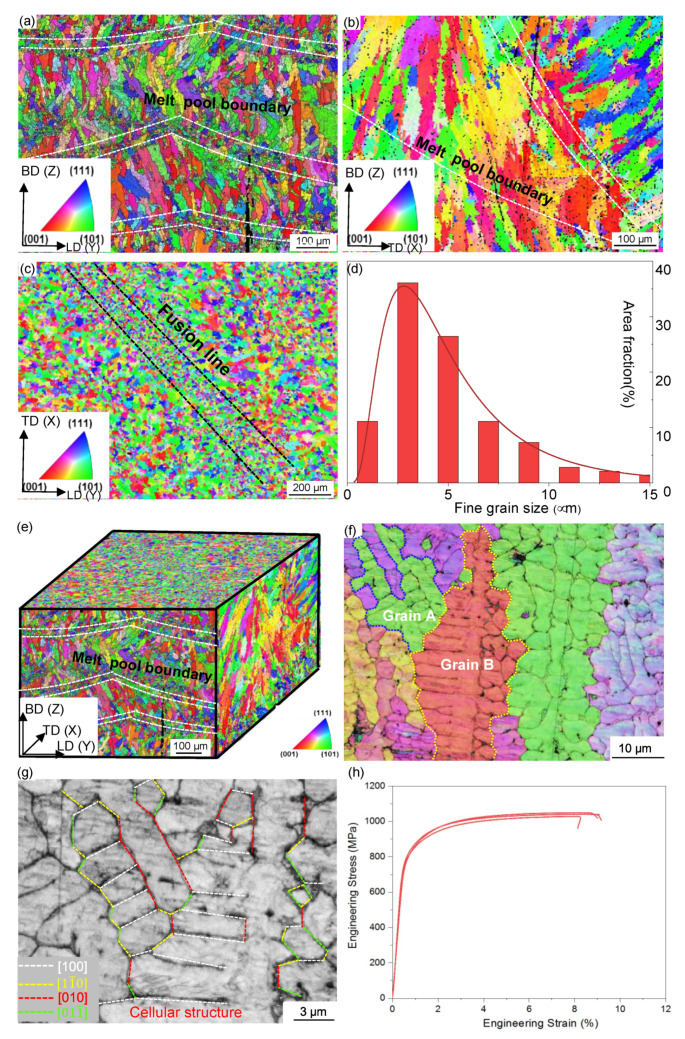
Microstructure and tension property of as-built 316L SS. (**a**–**c**) EBSD orientation maps in the LD-TD, BD-LD, and BD-TD plane. Here LD, TD, and BD stand for the longitudinal, transverse and build direction in turn. (**d**) The grain size distribution is obtained from the LD-TD surface. (**e**) The 3D mapping of grain microstructure. (**f**) The orientation map in BD-LD plane of the individual grains. Dash lines are used to identify grains A and B. (**g**) Band contrast map of the cellular structure and dash lines indicating cellular boundaries. (**h**) Uniaxial tensile stress–strain curves of as-built 316L SS.

We prepared 316L SS by LAM process, and its microstructure and mechanical property are shown in Figure 1. From the EBSD orientation maps in Figure 1a–c, we see both equiaxed and elongated columnar grains in planes composing two characteristics directions out of the longitudinal direction (LD), the transverse direction (TD), and the build directions (BD). The average grain size is around 3 μm viewed from the LD-TD plane (Figure 1d). The three-dimensional microstructures, viewed from different planes normal, are shown in Figure 1e. The EBSD orientation map along BD in Figure 1f shows typically structure of grain. Here, the boundaries of two grains A and B are highlighted. We show in Figure 1g the close-up view about the morphology and direction of cellular structures in the two grains. Dash lines indicating the cellular boundaries are aligned with either [100] or [110] crystalline direction in each grain and coincide with the thermal flow direction: thermal melting flow is associated with the minimum Young’s modulus direction or the axis of symmetry of the plane with minimum crystal surface energy [16]. We show in Figure 1h the engineering stress–strain curves of LAM 316L SSs, which have a yield strength of 850 MPa, together with an ultimate tensile strength of 1.05 GPa and a total elongation of 9%.

**Figure 2:**
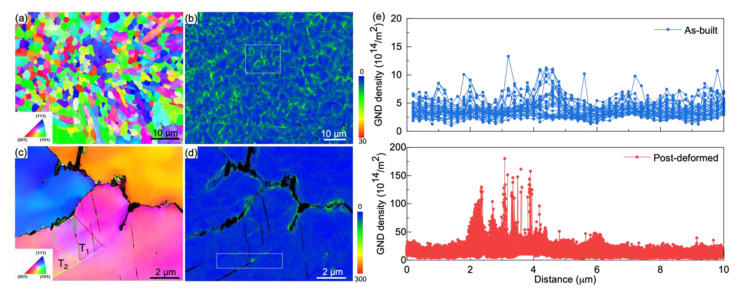
Microstructures of as-built and post-mortem samples. (**a**) The initial EBSD orientation map in the LD-TD plane. (**b**) Distribution of geometrically necessary dislocations. (**c**) The morphology of a post-mortem sample and (**d**) its GNDs distribution. (**e**) GNDs distribution profiles of the selected areas in (**b**,**d**), respectively.

To understand the role of the cellular boundary in deformation process, we compared geometrically necessary dislocations (GNDs) density in a sample between its as-built and deformed status, as shown in Figure 2. From the IPF (Figure 2a) and GNDs density distribution maps of the as-built sample, we derive the GNDs density within the bracket of (2~12) × 10^14^/m^2^. There is higher GNDs density in MCSs than that in interior, as evidently seen in Figure 2b. In the post-mortem sample, we observe hierarchical deformation twins, marked by T_1_ and T_2_. The GNDs density in the deformed sample ascends to the range of (2~18) × 10^15^/m^2^. Interestingly, abundant dislocation emissions take place at the junction of hierarchical twins and cellular boundaries, as shown in Figure 2c,d. As seen from Figure 2e, the GNDs density of the deformed sample is about one order of magnitude higher than its initial status.

**Figure 3:**
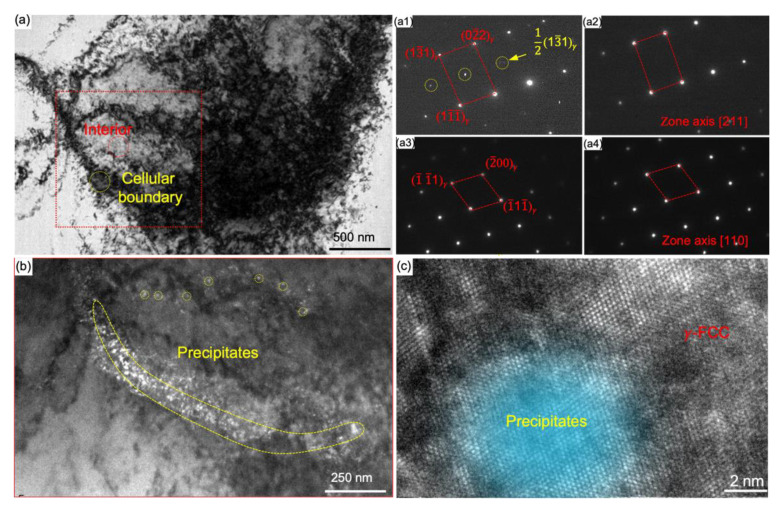
Morphology of precipitates in a MCS. (**a**) BF-TEM image of a MCS composing of cellular boundary (in yellow circle) and interior (in red circle). (**a1**–**a4**) The SADPs of cellular boundary (**a1**,**a3**) and interior (**a2**,**a4**). Taking from zone axis [112] (**a1**,**a2**) and [110] (**a3,a4**) respectively. (**b**) DF-TEM image highlighting dense precipitates corresponds to the region marked by the red box in (**a**). (**c**) HR-TEM image containing the matrix and precipitate in [110] direction, the marked region indicating the coherent precipitate in *γ*-FCC matrix.

We now apply selected area diffraction pattern (SADP) to distinguish the configurations of cellular boundary from interior. The bright field TEM (BF-TEM) image in Figure 3a shows that the MCS is composed of the cellular boundary (indicated in yellow) and the cellular interior (indicated in red). The SADP in the zone axis [112] indicates the extra super lattice spots in the 1/2 (311) crystalline plane (highlighted in yellow circle in Figure 3a1). Whereas the cellular interior in Figure 3a2 shows typical *γ* face cubic crystal (*γ*-FCC) structure in the same [112] zone axis, without additional diffraction pattern. When observing along the [110] zone axis, the diffraction patterns are the same for both cellular boundaries and interiors, as shown in Figure 3a3,a4. These observations imply that the coherent diffraction structure appears in (311) crystalline plane when observing along the [112] zone axis. We then take dark-field TEM (DF-TM) image using the extra reflections 1/2 (311) spot in yellow circles in Figure 3a1. The light-up region marked by yellow dash lines suggests nanoscale precipitates distribute densely along the cellular boundary from DF-TEM image (see in Figure 3b). We take the HR-TEM image (Figure 3c) containing both *γ*-FCC matrix and a precipitate in [110] zone axis. It demonstrates that the precipitates are coherent with the matrix. The *α* parameter measured for the matrix and precipitate is 0.3537 ± 0.002 and 0.3628 ± 0.003 respectively, with a misfit of about 2.5%.

**Figure 4:**
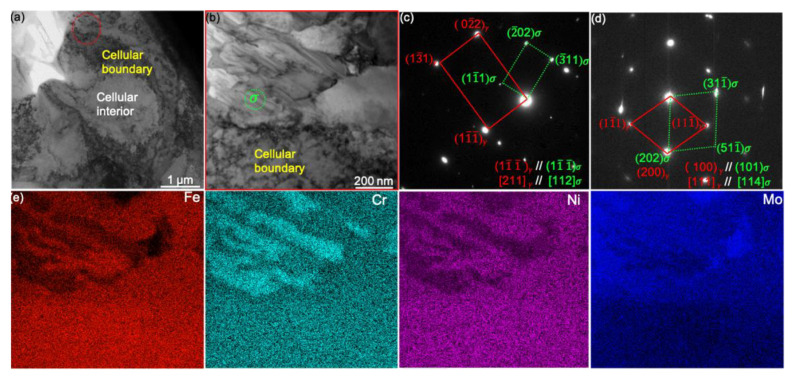
Lamellar-like *σ* phase nearby a MCS. (**a**) The BF-TEM image of *σ* phase. (**b**) A blow-up high-angle annular dark-field (HAADF) STEM image showing the lamellar structure. (**c**,**d**) The SADPs indicate the orientation relationship of *γ* matrix and *σ* phase along the zone axis of *γ* matrix: (**c**) [211] and (**d**) [111]. (**e**) The energy-dispersive spectroscopy (EDS) maps of Fe, Cr, Ni, and Mo distribution in the region shown in (**b**).

We also observed the *σ* phase next to the MCS, as shown in Figure 4a, with its blow-up shown in Figure 4b: the *σ* phase is composed of alternatively lamellar-like layers. The SADPs in Figure 4c,d suggest the *σ* phase owns tetragonal crystal structure and follows the orientation relationship [112]*σ*//[211]*γ*, [114]*σ*//[111]*γ* with the *γ*-FCC matrix. The energy-dispersive spectroscopy (EDS) map in Figure 4e illustrates that *σ* phase is composed of alternative Cr or Fe (Ni) rich lamellas. As reported, the formation of *σ* phase in austenite steels is promoted by enrichment of Cr or Mo [33] and the thermal cycling stress on the cellular boundary [34]. In the current work, the generation of *σ* phase seems to be associated with the chemical fluctuation around the cellular boundary.

The origins of coherent precipitates and *σ* phase revealed here appear to be related to the decomposition process due to concentrations of Cr, Ni, or Mo solutes in LAM process. We suspect that the “spinodal decomposition” is mainly account for the phase decomposition in multicomponent as-built 316L SS. Based on the non-equilibrium solidification theory [35,36,37,38], any chemical fluctuations may enable the “uphill diffusion” and “spinodal decomposition” [35,37,38,39]. This phenomenon has been proven for precipitation in high-entropy alloys due to the “sluggish diffusion” of solutes [9,39]. In terms of the as-built alloys by LAM, the “spinodal decomposition” has the potential to take place due to “insufficient diffusion” by high cooling rate. During the non-equilibrium solidification process, the “spinodal decomposition” is plausible as the temperature gradients induce the vibration entropy [18,40,41,42]. Chemical fluctuation owing to solutes segregation provides the driving force for spinodal and transient spinodal phase separation at the cellular boundary in as-built 316L SS. Therefore, the ordered coherent phase prevails along the cellular boundary. Furthermore, the elastically soft direction is favorable for spinodal growth so that the coherency strain is minimized by decomposition [36]. It explains why majority of the precipitates are aligned with [100] crystalline direction. Albeit this phenomenon has been broadly seen in aged heat treatment steels [18,33,34,35,36,37,38], it is firstly proposed in the as-built 316L SS.

**Figure 5:**
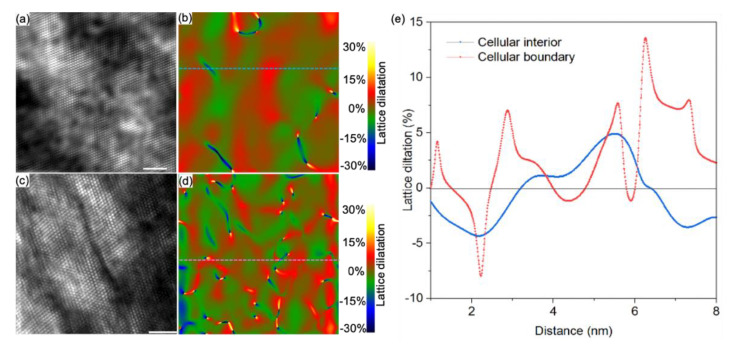
Dislocation structures in an MCS. (**a**) IFFT image of cellular interior taking along the [110] zone axis, (**b**) its GPA map showing dislocation cores with (111) frequency. (**c**) IFFT images of the cellular boundary, and (**d**) its GPA map. The color legend for GPA map indicates *ε_xy_* induced by dislocations. (**e**) The local *ε_xy_* corresponding to dash lines marked in (**b**,**d**). The scale bar is 2 nm.

Apart from configurations, we further explore the dislocation arrangements at a MCS. The HR-TEM images and geometric phase analysis (GPA) maps in Figure 5 help to distinguish dislocation structures and lattice strain field in the cellular boundary from the interior. The Inverse fast Fourier transformation (IFFT) image (Figure 5a) and its *ε_xy_* lattice strain map by GPA using (111) frequency (Figure 5b) indicate that limited lattice distortions and isolated dislocation appear in the interior. Contrastively, dislocation dipoles and entanglements, containing stacking faults, are operative at cellular boundary as identified from the IFFT image and GPA map in Figure 5c and d, respectively. The lattice dilatations profile in Figure 5e confirms the cellular boundary experiences more cycles of compression and tension strain than that in the interior. It seems that thermal and solutes contribute to the accumulation of lattice dilatations during solidification in as-built alloys [27,43,44]. Chemical composition fluctuation is likely the governing reason for such dislocation structures and lattice strain difference between the cellular boundary and interior. Hence, the co-existence of dislocation and precipitates in MCS is the stark feature in the as-built 316L SS.

**Figure 6:**
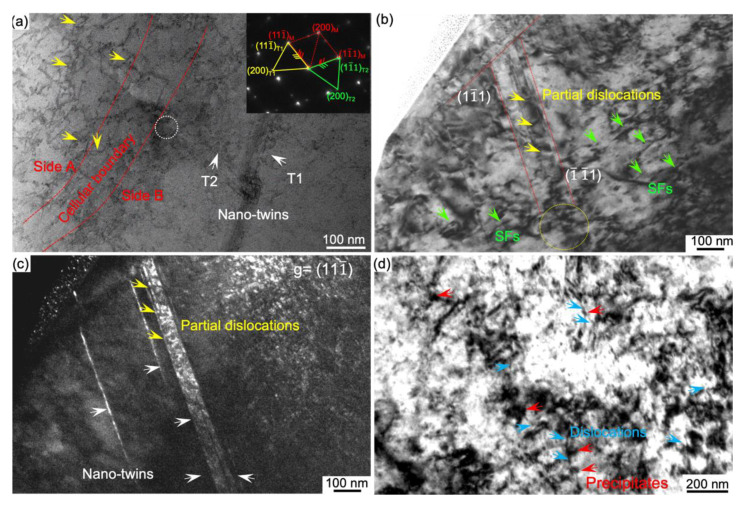
Post-mortem TEM observations. (**a**) BF-TEM image showing twin interactions with the MCS. Hierarchical nanotwins and the cellular boundary are indicated in white arrows and red lines respectively. The yellow arrows and the white circle indicate dislocation loops and dislocation piling up region respectively. (**b**) The BF-TEM image showing SFs (in green arrows) and partial dislocations (in yellow arrows). (**c**) The DF-TEM image taken with g = $11\bar{1}$. (**d**) Precipitates in a MCS (marked as red arrows) are cut through by dislocations indicated as blue arrows.

To investigate the role of MCS in plastic deformation, we further perform TEM observation of the post-mortem sample, as illustrated in Figure 6. We prepare the TEM lamellar in the interested area including the interaction of MCS with hierarchical twins, as mentioned in Figure 2d. The SADP inserted in Figure 6a indicate the hierarchical twins are operative. The BF-TEM image in Figure 6a shows that tangled and looped dislocations (yellow arrows) prevail near side ‘A’. Usually, the dislocation loops around side ‘A’ of cellular boundary are induced by Frank dislocations, which are associated with local supersaturation of vacancies at high cooling rate [45].When a twin penetrates through side ‘A’ of cellular boundary, a dislocation reaction follows,

(1) $$\frac{a}{6}\left[\;\bar{2}1\bar{1}\;\right]+\frac{a}{3}\left[11\bar{1}\right]\to \frac{a}{2}\left[01\bar{1}\right]$$

As twinning propagates from side ‘A’ to side ‘B’, dislocations multiply and pile up closely to side ‘B’, as seen from white circle in BF-TEM image (Figure 6b). It also shows that a large density of nano-spaced stacking faults (green arrows) and partial dislocations (yellow arrows) arise from massive dislocation dissociations. Subsequently, more partial dislocations prevail at side ‘B’, accompanied with nanotwins, as seen in DF-TEM image (Figure 6c). It appears that the motion of dislocations is hindered but not fully stopped by the cellular boundary, which is in line with the in-situ observation of twinning interactions with the cellular boundary [21,22]. Overall, it implies that the threshold of nanotwins nucleation can be decreased by the variation of SFE at the MCS, which is commonly seen from the twinning nucleation theory [46]. Additionally, the dislocation slip prevails in the MCS during deformation as well. Figure 6d shows that the coherent precipitates (in red arrows) can be sheared by dislocations, as indicated in blue arrows. After shearing, the antiphase boundary (APB) can be created on the dislocation gliding plane, consequently, the ordering hardening is possible to be the source for strengthening [47,48,49].

**Figure 7:**
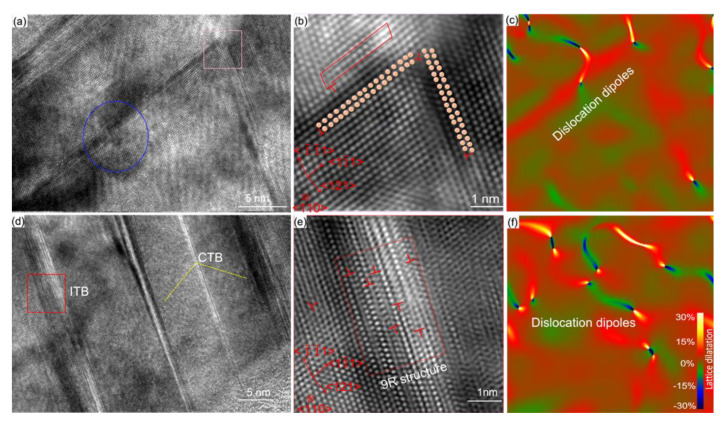
HR-TEM images close to an MCS. (**a**) HR-TEM of the ‘V’-shaped configuration of SF. The pink and blue area indicate Lomer–Cottrell locks and APB. (**b**) A close-up IFFT of the pink box in (**a**,**c**) GPA map with (200) frequency showing dislocation structure. (**d**) HR-TEM images of twins. The yellow and red box indicate the CTB and ITB respectively. (**e**) The IFFT images of ITB. (**f**) The corresponding GPA map.

The HR-TEM image (Figure 7) adjacent to an MCS shows that coherent precipitates are sheared by edge dislocations on ($1\bar{1}1$) crystalline plane, as indicated in the blue circle; it further confirms the APB forms when dislocations cutting through the coherent precipitates. Interestingly, we observe the ‘V-shaped’ dislocation network (boxed in pink in Figure 7a) as well. The close-up IFFT image of the ‘V-shaped’ dislocation in Figure 7a demonstrates edge dislocations glide on ($1\bar{1}1$) and ($\bar{1}\bar{1}1$) crystalline planes, as detailed in Figure 7b. The dislocations dissociate as follows

(2) $$\frac{a}{2}\left[110\right]\;\to \frac{a}{6}\left[121\right]+\frac{a}{6}\left[21\bar{1}\right]$$

(3) $$\frac{a}{2}\left[\bar{1}0\bar{1}\right]\to \frac{a}{6}\left[\bar{2}1\bar{1}\right]+\frac{a}{6}\left[\bar{1}\bar{1}\bar{2}\right]$$

Burgers vector in red in Figure 7b indicates that the immobile stair-rod dislocations are generated via $\frac{a}{6}\left[121\right]+\frac{a}{6}\left[\bar{1}\bar{1}\bar{2}\right]\to \frac{a}{6}\left[01\bar{1}\right]$, which are known as Lomer–Cottrell locks [50,51]. The Lomer–Cottrell lock is possible to enhance the strain hardening as the dislocation reactions [51]. Figure 7c illustrates that the dislocation cores and the lattice strain field *ε_xy_* of Lomer–Cottrell locks are derived from its GPA map with (200) frequency. Furthermore, the heterogeneous MCS promotes the nanotwins to nucleate, accompanied with coherent twin boundary (CTB) and incoherent twin boundary (ITB), as marked in Figure 7d. The corresponding IFFT images suggest partial dislocations prevail, as shown in Figure 7e, together with the corresponding GPA map of ITB (Figure 7f). The ITB belongs to $\sum^{\;}3\left\{112\right\}$ with 9R structures twinning behavior, as commonly seen in nanocrystalline alloys [52,53]. It further suggests that the reduced stacking fault contribute to the twinning nucleation and dislocation dissociation when hierarchical twin interacts with the MCS [46]. As expected, the dislocation structures are associated with variation of SFE due to the local chemical fluctuation in the MCS [11,15,25].

**Figure 8:**
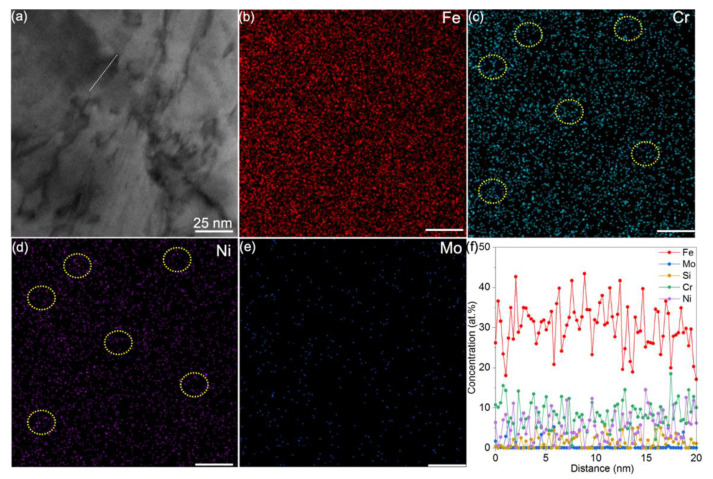
Microstructure and the EDS composition map. (**a**) The HAADF-STEM image adjacent to a MCS in Figure 5b. (**b**–**e**) The corresponding EDS maps for individual elements of Fe, Cr, Ni, and Mo, in turn. (**f**) Composition profile along the white line in (**a**), showing local chemical fluctuation.

As mentioned above, dislocation dissociations are linked to SFE variations, which can be tailored by local chemical environment. We perform the chemical distribution analysis in the region around the MCS, as shown in HAADF-STEM (Figure 8a). The composition map in Figure 8b–e displays the solutes Fe, Cr, Ni, and Mo distribution respectively. The circled region in Figure 8c,d indicate the solutes Cr and Ni clustered area. The line analysis across the boundary suggests that Cr shares a similar degree of inhomogeneity with Ni, whereas Fe has the opposite fluctuation tendency, as shown in Figure 8f. Meanwhile, the atomic ratio of the Fe, Ni, and Cr is (2~4):1:1, therefore, the ordered precipitate mentioned in Figure 3 is assumed to be Fe_2_CrNi as the similar atomic ratio. This ordered precipitate has been found in aged Fe-Cr-Ni austenite steels [54,55,56], while it is newly reported here in the as-built 316L SS.

According to the documented SFE evaluations in concentrated alloys [23,24,25,26,57], an empirical equation is used to estimate the SFE variation following [57],

(4) SFE=2.8 Ni(wt.%)+0.39 Cr(wt.%)+2.2 Mo(wt.%)−2.0 Si(wt.%)−4.0

**Figure 9:**
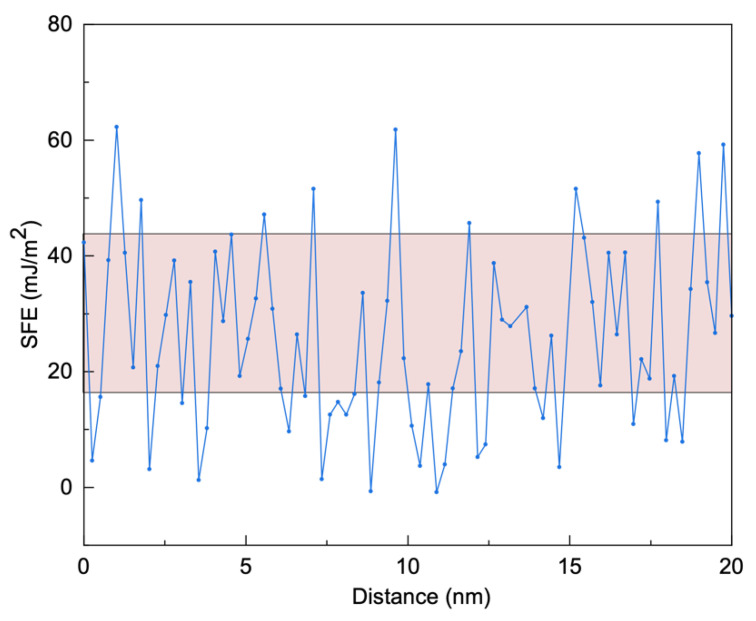
SFE profile calculated based on an empirical formula. The marked range of the SFE data from the reference [25,57].

We plot the SFE profile as a function of distance in Figure 9 based on the chemical fluctuation in Figure 8f. We reveal that the SFE energy is not constant due to chemical fluctuation in the MCS, in contrast to the single-valued SFE in conventional crystalline counterparts. This is in line with the commonly studied concentrated alloys [22,23,24,55]. The published SFE data of 316L SS are in the range of (12.9~42) mJ/m^2^, which are included as the red background in Figure 9. The variation of SFE strongly influences plastic deformation mechanisms, in particular for dislocation multiplication [25,58]. As a result, the SFE variation in heterogeneous MCS is crucial for tailoring mechanical properties.

Local chemical variation gives rise to lattice distortions at the atomic scale resulting from the SFE variations. Therefore, the dislocation line travelling in a field of solutes will be wavy in nature [59]. The bowing of the dislocation lines occurs at the cost of extra line energy compared to an otherwise straight dislocation segment. These additional nano-disturbances of SFE account for dislocation strengthening as well.

## 4. Conclusions

Metastable cellular structures (MCSs), as a result of the LAM process, are pivotal to the deformation behavior of concentrated alloys. Taking as-built 316L SS as a model case, we conducted a systematic investigation on the structure of MCSs and mapped the structures to deformation mechanisms. The following observations may be significant for later research about this aspect:

(1) We revealed that highly ordered coherent precipitates present along the MCS resulting from spinodal decomposition by local chemical fluctuation. The co-existences of coherent precipitates and high density of tangled dislocations at MCS serve as walls for extra hardening.
(2) We demonstrated the existence of local chemical fluctuation in MCSs and suspect the induced variation in stacking fault energy is an important factor for ductility enhancement.
(3) We proposed that the synergistic contributions from the ordering strengthening by coherent precipitates and dislocations strengthening by high density of tangled dislocations in the heterogeneous MCS lead to the extra strain hardening in as-built 316L SS.

These findings provide a sound foundation for understanding of the local chemical in MCSs to tune precipitates and dislocation structures with the aid of LAM process.

## Data Availability

Data is contained within the article.
